# Tangeretin’s Anti-apoptotic Signaling Mechanisms in Oral Cancer Cells: In Vitro Anti-cancer Activity

**DOI:** 10.7759/cureus.47452

**Published:** 2023-10-22

**Authors:** Venkatakarthikeswari GV, Priyadharshini Ranganathan, Sinduja Palati

**Affiliations:** 1 Pathology, Saveetha Dental College and Hospitals, Saveetha Institute of Medical and Technical Sciences, Saveetha University, Chennai, IND; 2 Oral and Maxillofacial Pathology, Saveetha Dental College and Hospitals, Saveetha Institute of Medical and Technical Sciences, Saveetha University, Chennai, IND

**Keywords:** apoptosis, tangeretin, oral cancer, cell line, anti-cancer activity

## Abstract

Introduction

Citrus fruit peels contain *Tangeretin*, a natural chemical flavonoid that reinforces plant cell walls and serves as a defense mechanism. Apoptosis, growth inhibition, anti-oxidant, anti-diabetic, and anti-cancer activities are only a few of its many qualities. *Tangeretin*'s principal function is to shield healthy cells or tissues from the harmful effects of chemotherapy. The purpose of this study was to investigate the apoptotic activity of *Tangeretin*'s impact on KB (oral cancer cells) cell lines.

Materials and method

This study employed *Tangeritin,* in investigating its effects on oral cancer cells. Oral cancer cells were cultured in Dulbecco's modified Eagle's medium (DMEM), with 10% fetal bovine serum at 37°C in a 5% CO_2_ environment. Cell viability was assessed by seeding oral cancer cells in 96-well plates, exposing them to varying *Tangeritin *concentrations (50 µM, 100 µM, and 200 µM) with growth inhibition of KB cell viability in 3-(4,5-dimethylthiazol-2-yl)-2,5-diphenyltetrazolium bromide (MTT) assay and morphological changes in cells were observed under an inverted light microscope at 10x magnification. The results were reported as mean ± standard error mean (SEM) using one-way analysis of variance* *through IBM SPSS Statistics for Windows, Version 23 (Released 2015; IBM Corp., Armonk, New York, United States).

Result

MTT assay showed a significant reduction in KB cell viability when treated with *Tangeretin*. With a* *significant decrease in mRNA levels of the anti-apoptotic genes Bcl-2 and Bcl-xL. At 50 µM, 100 µM, and 200 µM, the levels of Bcl-2 were 0.85 ± 0.09, 0.62 ± 0.05, and 0.67 ± 0.05, respectively. Similarly, the mRNA expression of Bcl-xL was 0.82 ± 0.07 for 50 µM, 0.7 ± 0.06 for 100 µM, and 0.77 ± 0.06for 200 µM. The mRNA expression levels of Bax were 1.1 ± 0.09 for 50 µM, 1.4 ± 0.12for 100 µM, and 1.3 ± 0.11 for 200 µM, respectively.

Conclusion

*Tangeretin *showed a promising apoptotic activity in KB cells suggesting its utility as an anti-cancer compound. It prevented the growth and proliferation of cancer cells by acting on pro-apoptotic and anti-apoptotic genes. However, this conclusion is mostly based on the in vitro study. Therefore in vivo animal studies were needed to confirm the findings.

## Introduction

*Tangeretin *is a polymethoxylated flavone (PMF), primarily found in the peels of citrus fruits like tangerines, oranges, and mandarins. It serves as a protective mechanism in plants by strengthening their cell walls. This compound is abundant in citrus fruits and offers various beneficial effects, including apoptosis induction, growth inhibition, immune system enhancement, anti-angiogenic properties, and anti-cancer effects [1]. Extensive research, both in vitro and in vivo, supports its hypothesized bioactivities, such as anti-oxidant, neuroprotection, anti-microbial, anti-inflammatory, and efflux pump inhibition [2].

*Tangeretin*'s role in inhibiting cell growth in the G1 phase by promoting the production of cyclin-dependent kinase inhibitors, specifically p27 and p21, has been demonstrated [3]. It has also been explored as a treatment for oxidative stress-related effects in cancer patients, such as DNA mutations and aberrant cell growth. In experiments with cancer cells, *Tangeretin* administration resulted in cell cycle arrest at G2/M/G1/S phases, reduced cell migration, and decreased cell proliferation [4].

*Tangeretin* has shown anti-cancer properties in breast cancer cells (MCF-7) by inhibiting cell growth and promoting apoptosis through mitochondrial disruption [5]. It also inhibits cancer cell migration and proliferation by causing chromatin condensation, apoptotic body formation, reduction in mitochondrial membrane potential (MMP), increased pro-apoptotic proteins, and nuclear shrinkage. Importantly, *Tangeretin* protects non-cancerous cells and tissues from damage caused by chemotherapy treatments [6].

Numerous in vitro and in vivo experiments consistently demonstrate *Tangeretin*'s ability to inhibit cancer cell growth and progression, highlighting its potential as an anti-cancer agent. However, there is a relative scarcity of research on the detailed mechanisms and efficacy of its anti-cancer properties [7]. This study aimed to evaluate the apoptotic activity of *Tangeretin* in oral cancer cells.

## Materials and methods

Procurement and culture of oral cancer cells and chemicals

Chemicals utilized for the study were described in Table 1. Oral cancer cell line was obtained from National Centre for Cell Science (NCCS), Pune, India. Cancer cells were grown in a Minimal Essential Medium (10% fetal bovine serum at 37˚C under 5% CO_2_).

**Table 1:** Details of the chemicals used in the experiments carried out in the study. EDTA: Ethylenediaminetetraacetic acid; FBS: Fetal bovine serum; DMEM: Dulbecco's modified Eagle's medium; PBS: Phosphate buffered saline; PCR: Polymerase chain reaction; MTT: 3-(4,5-dimethylthiazol-2-yl)-2,5-diphenyltetrazolium bromide

Chemicals	Manufacturer details and location
Tangeretin	Sigma-Aldrich Chemicals Private Limited, Bangalore, India
Trypsin-EDTA	Gibco Enterprises, Ontario, Canada
FBS	Gibco Enterprises, Ontario, Canada
antibiotics-antimycotics	Gibco Enterprises, Ontario, Canada
DMEM	Gibco Enterprises, Ontario, Canada
PBS	Gibco Enterprises, Ontario, Canada
JC-1 (5,5,6,6-tetrachloro-1,1,3,3-tetraethylbenzimidazolylcarbocyanine iodide)	Invitrogen Corporation, Carlsbad, USA
Real-time PCR kit (MESA Green)	Invitrogen Corporation, Carlsbad, USA
MTT	Sigma-Aldrich Chemicals Private Limited, Bangalore, India

MTT assay, real-time polymerase chain reaction, and gene analysis

Oral cancer cells were introduced into 96-well plates at a concentration of 5x10⁵ cells per well and allowed to adhere overnight. Following this initial attachment, the cells were exposed to varying concentrations of *Tangeritin* in triplicate and then cultured at 37˚C in a CO_2_-rich environment for 24 hours. Next, we introduced 3-(4,5-dimethylthiazol-2-yl)-2,5-diphenyltetrazolium bromide (MTT) into each well and continued the incubation for an additional four hours at 37˚C. To dissolve the formazan compounds created from MTT, the cells were resuspended in 200 µl of dimethyl sulfoxide, and the optical density (OD) was measured using a spectrometer at a wavelength of 570 nm. This entire procedure was independently repeated three times. Subsequently, the mean OD ± standard deviation (SD) was computed for each set of replicates. The entire process was carried out in triplicate. To calculate the inhibition rate of cell growth, the following equation was employed: % growth inhibition = (1 - ODextract treated)/ODnegative control x 100 [8].

Real-time polymerase chain reaction (PCR) is used to measure the amounts of mRNA expression during gene expression studies. The TRI Reagent® (Sigma) was used to separate the total RNA. Using a commercial SuperScript™ III First-Strand cDNA Synthesis Kit (Invitrogen Corporation, Carlsbad, USA), total RNA (2g) from each sample was reverse transcribed in accordance with the manufacturer's instructions. Stratagene's MX3000p PCR machine (Stratagene California, San Diego, USA) was used to conduct real-time PCR. The MESA Green PCR Master Mix (Invitrogen Corporation, Carlsbad, USA), which includes SYBR green dye and all of the PCR components, was used to conduct the reaction. Melting curve analysis for each pair of primers was used to determine the specificity of the amplified product. Utilizing CFX Manager Version 2.1 (Bio-Rad Laboratories, Inc., Hercules, USA), the data were examined using the comparative CT approach, and the fold change was determined using the 2CT method.

Statistical analysis

Cell viability at different concentrations of *Tangeretin* was recorded and mean ± SEM was calculated. Similarly fold change for Bcl-2, Bcl-xL, and Bax were obtained and mean ± SEM was derived. Further cell viability between three different concentrations of *Tangeretin *was compared using a one-way analysis of variance to analyze any significant difference in fold change at various concentrations.

## Results

**Figure 1:**
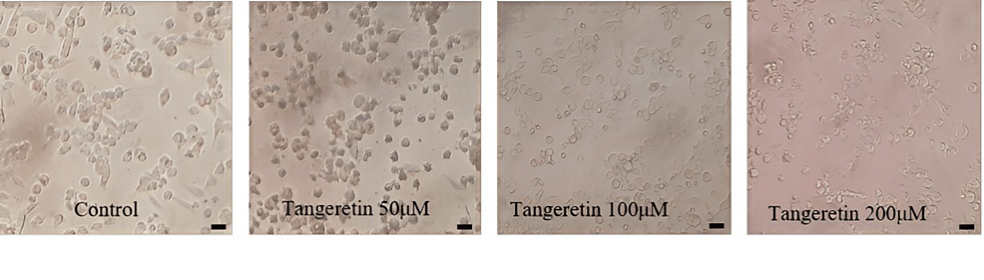
Tangeretin was treated with KB cells (50 µM, 100 µM, and 200 µM), and morphological changes in cells were observed under an inverted light microscope at 10x magnification.

**Figure 2:**
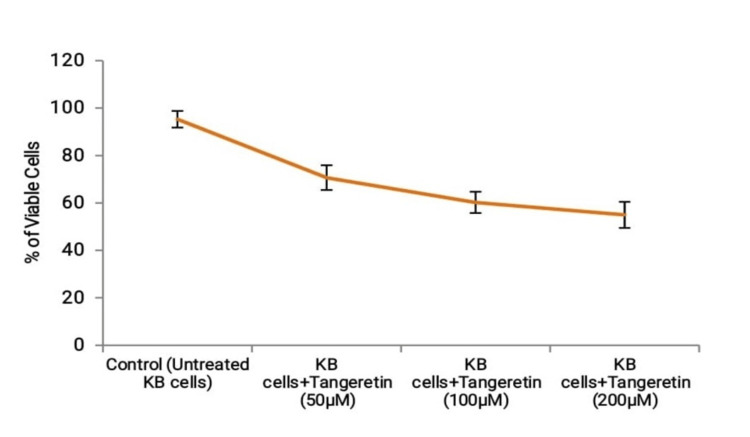
Cell viability assessment using MTT assay. MTT: 3-(4,5-dimethylthiazol-2-yl)-2,5-diphenyltetrazolium bromide

*Tangeretin*'s viability was evaluated through an MTT assay to investigate its impact. After 48 hours of treatment, the effectiveness of *Tangeretin *exhibited a significant reduction in KB (oral cancer cells) cell viability caused by the treatment (Figures 1, 2). These findings showed the potent cytotoxic influence of *Tangeretin* on KB cells.

**Figure 3:**
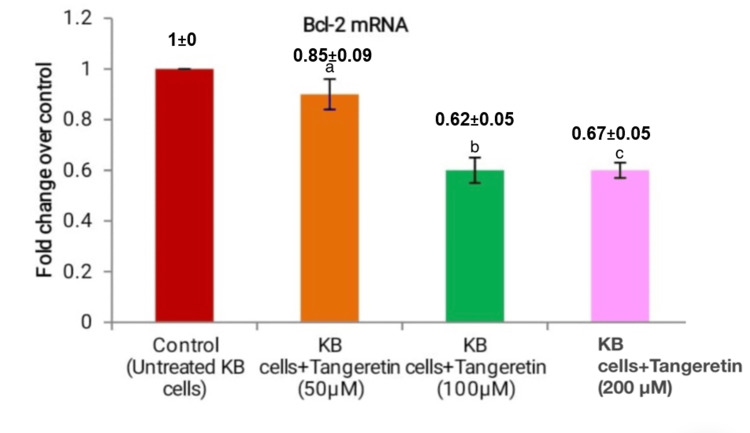
Tangeretin concentration at various micromoles in the KB cell line was indicated on the X-axis, and the fold change in Bcl-2 mRNA expression was indicated on the Y-axis. a) 50 µM of Tangeretin in KB cell line, b) 100 µM of Tangeretin, and c) 200 µM of Tangeretin treated KB cells. There was a significant decrease with a rise in Tangeretin concentration, inhibition of cancer cells was seen from 100 µM concentration indicating maximum anti-apoptotic activity.

**Figure 4:**
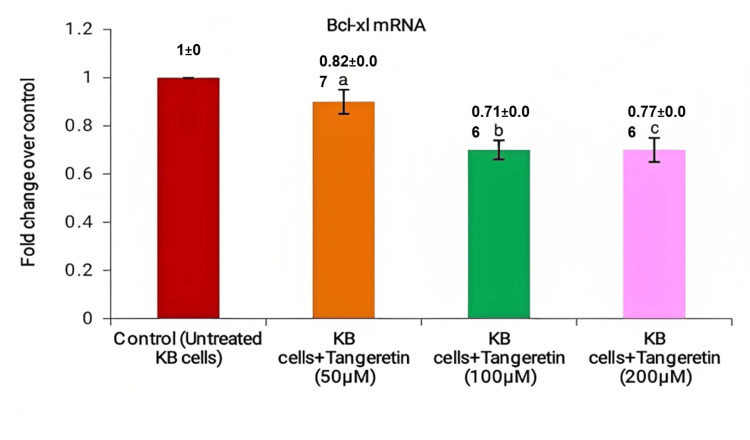
Tangeretin concentration at various micromoles in the KB cell line was indicated on the X-axis, and the fold change in Bcl-xL mRNA expression was indicated on the Y-axis. a) 50 μM of Tangeretin in KB cell line, b) 100 μM of Tangeretin, and c) 200 μM of Tangeretin treated KB cells. There was a significant decrease with a rise in Tangeretin concentration, maximum inhibition is seen from 100 μM concentration indicating maximum anti-apoptotic activity.

*Tangeretin* at various concentrations in KB cell line, was used to assess the mRNA expression levels of Bcl-2, Bcl-xL, and Bax using real-time PCR. In comparison to the control group, *Tangeretin *demonstrated a significant decrease in mRNA levels of the anti-apoptotic genes Bcl-2 and Bcl-xL. At 50 µM, 100 µM, and 200 µM, the levels of Bcl-2 were 0.85±0.09, 0.62±0.05, and 0.67±0.05 (Figure 3). Similarly, the mRNA expression of Bcl-xL was 0.82±0.07 for 50 µM, 0.71±0.06 for 100 µM, and 0.77±0.06 for 200 µM (Figure 4).

**Figure 5:**
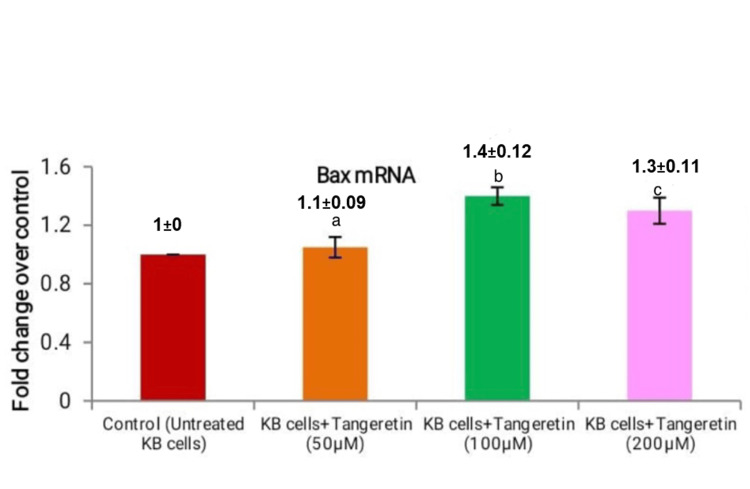
Tangeretin concentration at various micromoles in the KB cell line was indicated on the X-axis, and the fold change in Bax mRNA expression was indicated on the Y-axis. a) 50 μM of Tangeretin in KB cell line, b) 100 μM of Tangeretin, and c) 200 μM treated PC-3 cells. An increase in Tangeretin concentration levels showed a considerable rise in pro-apoptotic activity.

Moreover, the administration of *Tangeretin *resulted in the suppression of pro-apoptotic gene Bax expression. The mRNA expression levels of Bax were as follows: 1.1±0.09 for 50 µM, 1.4±0.12 for 100 µM, and 1.3±0.11 for 200 µM (Figure 5).

## Discussion

*Tangeretin *as a natural flavonoid has good anti-cancer activity, with effective anti-apoptotic properties at higher concentrations. The results demonstrated a 50% reduction in viable cells at higher concentrations of *Tangeretin*. According to the current study, *Tangeretin* alone exhibits strong anti-cancer effects through pro-apoptotic mechanisms. According to a recent study, *Tangeretin* exerts growth-inhibitory activity by suppressing Cdk2 and Cdk4 kinase as well as by elevating p21 and p27 levels. In another study, it reduced COLO205 cell proliferation by arresting cell cycle progression in the G1 phase [9]. Similarly, Chen et al. reported tumor suppression activity by *Tangeretin* in human MCF-7/6 breast cancer cells cultured in female nude mice [10]. Further, *Tangeretin* showed better tumor suppression activity at low doses compared to low-dose cisplatin. This was due to the down-regulation of the PI3K/Akt signaling pathway [11]. These results indicate cytotoxic activity of *Tangeretin* was through apoptotic pathways through apoptotic genes such as Bcl-2, Bcl-xL, Bax, etc.

During programmed cell death, the upregulation of the anti-apoptotic gene Bcl-2 activates tumor suppressor genes through the mitochondria, thereby inducing caspase-9 activation, ultimately leading to apoptosis. However, in the context of carcinogenesis, cancer cells evade apoptosis and instead undergo uncontrolled cellular proliferation. Notably, the current investigation has revealed that *Tangeretin *can effectively target this pathway, leading to the induction of apoptosis. This is substantiated by the observed reduction in Bcl-2 expression levels within the study. These findings align with the work of Zheng et al., who demonstrated that *Tangeretin* disrupts hepatic cancer cell proliferation and migration in HepG2 cells by interfering with the Bcl-2 pathway, causing cell cycle arrest in the M/G2 phase [12]. Similarly, Arivazhagan et al. conducted research on mammary cancer in rats and observed that *Tangeretin *induces G1/S cell cycle arrest through the upregulation of p21 and p53 [13]. Furthermore, a study by Dong et al. on a gastric cancer cell line concluded that the upregulation of Bcl-2 primarily promotes apoptosis through mitochondrial dysfunction mediated by p53 and FasL/Fas signaling [14].

Similarly, Bcl-xL plays a regulatory role in the Akt/PI3K signaling pathway when exposed to* Tangeretin *in cancer cells. Guo et al. observed that *Tangeretin* induces DNA fragmentation by inhibiting anti-apoptotic genes like Bcl-xL, particularly in prostate cancer [15]. Study findings by Das et al. in the context of brain cancer elucidate how *Tangeretin *contributes to the downregulation of Bcl-xL, thereby promoting apoptosis [16]. In the present study, conducted on KB oral cancer cells, we found that increasing *Tangeretin* concentration leads to the inhibition of Bcl-xL expression. As previously mentioned, *Tangeretin* exerts its influence by inhibiting the expression of both Bcl-2 and Bcl-xL genes, consequently promoting the expression of the pro-apoptotic Bax gene. This multifaceted mechanism involves various pathways, including JNK, Akt/PI3K, p27, p53, and cyclins, and ultimately culminates in the activation of caspase 3, promoting apoptosis as outlined in previous studies [14,16,17]. Previous research has provided evidence of TNF-alpha's ability to increase the expression of the anti-apoptotic Bcl-2 protein in individuals afflicted with both high blood pressure [18] and oral cancer [19]. These results clearly highlight the utility of *Tangeretin *as a potential anti-cancer compound.

Limitations

Although *Tangeretin* showed potential apoptotic activity. The results were based on the in vitro study, based on the apoptotic gene expression. However, the study did not investigate the cell signaling pathway. Further, *Tangeretin* was a crude extract therefore specific compound for its apoptotic activity couldn't be ascertained.

## Conclusions

*Tangeretin* showed a promising apoptotic activity in KB cells suggesting its utility as an anti-cancer compound. It prevented the growth and proliferation of cancer cells by acting on pro-apoptotic and anti-apoptotic genes. However, this conclusion is mostly based on the in vitro study. Therefore in vivo animal studies were needed to confirm the findings.
